# Synchronisation between contrast media administration and caudocranial scan direction increases visualisation of altered coronary artery blood flow in patients presenting with dual left anterior descending coronary artery

**DOI:** 10.1259/bjrcr.20150500

**Published:** 2017-03-10

**Authors:** Charbel Saade, Fadi El-Merhi, Bassam El-Ashkar, Maha Mohamad, Ali Haydar, Antione Abchee

**Affiliations:** ^1^Department of Diagnostic Radiology, American University of Beirut Medical Center, Beirut, Lebanon; ^2^Department of Cardiology, American University of Beirut Medical Center, Beirut, Lebanon

## Abstract

Coronary CT angiography (CCTA) has the advantage over invasive coronary angiography in that its non-invasive nature and minimal risk on patients. CCTA enables accurate assessment of the entire heart, coronary artery system and thorax, displaying three-dimensional information about the spatial relations of the anomalous vessels and surrounding intraluminal and extraluminal anatomy, and thereby contributing clinically important prognostic information. Dual left anterior descending (LAD) coronary artery consists of of two LAD arteries within the anterior interventricular sulcus (AIVS). Type 4 is infrequently reported subtype and differs from the others, with a long LAD originating from the right coronary artery (Mercado, A., Johnson Jr, G., Calver, D., & Sokol, R. J. (1989). Cocaine, pregnancy, and postpartum intracerebral hemorrhage. *Obstetrics & Gynecology*, *73*(3, Part 2), 467-468. and the short LAD originating from the left main coronary artery. However, the radiological features between the short LAD and septal coronary arteries remain a controversy, with the latter being determined by CCTA. We present a case report based on short LAD terminating proximally in the AIVS and the long LAD originating from the RCA and terminating into the distal AIVS with the later having a long septal travelling parallel to the long LAD.

In the vast majority of people, the LAD coronary artery branches out of the left main coronary artery (LMCA) shaft and courses along the anterior interventricular groove (AIVG) all the way toward the apex of the heart. The presence of two LAD coronary arteries, referred to as the dual LAD anomaly, is a very uncommon occurrence in which there are two LAD arteries. Different types of dual LAD circulation have been characterized based on the origin and course of a short and a long LAD.

Spindola-Franco et al^2^ have initially described and characterized the first four types of dual LAD based on the origin and course of both a short and a long branch of the LAD (Figure 1). Until recently six types of the dual LAD circulation have already been described and which all fit under the umbrella of the dual LAD anomaly. A novel pattern (Type 7) was very recently reported in which the short LAD originates proximally and separately from the LMCA which then bifurcates into a left circumflex and a long LAD.^3,4^

**Figure 1.**
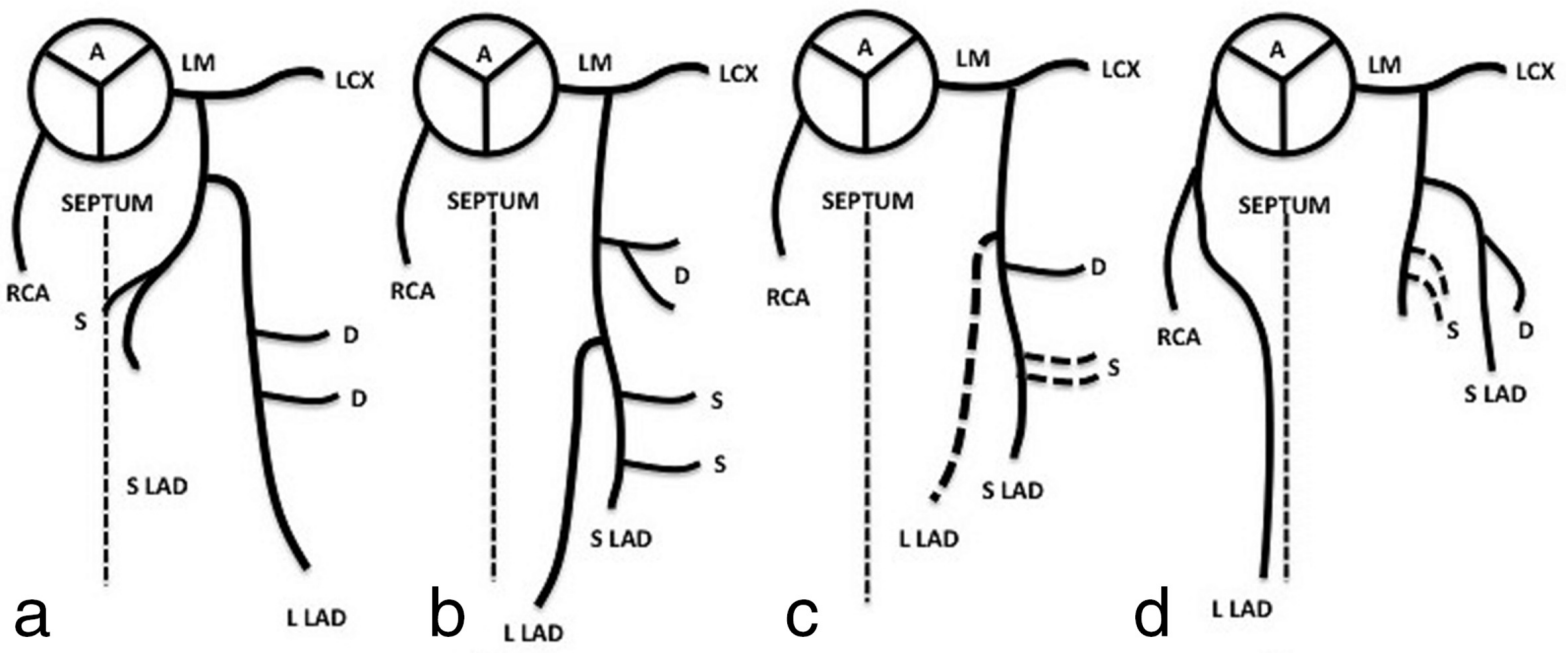
(a–d)schematic diagrams demonstrate four types of dual LAD. The first four subtypes were described by Spindola-Franco et al.^2^ Dashed lines indicate the AVIS septum. A, ascending aorta; AVIS, anterior interventricular sulcus; D, diagona1 coronary artery; LCX, left circumflex artery; LM, left main coronary artery; RCA, right coronary artery; S, septal coronary artery; S LAD, short left anterior descending artery; L LAD, long left anterior descending artery; LCX, left circumflex artery.

## Case presentation

**Figure 2.**
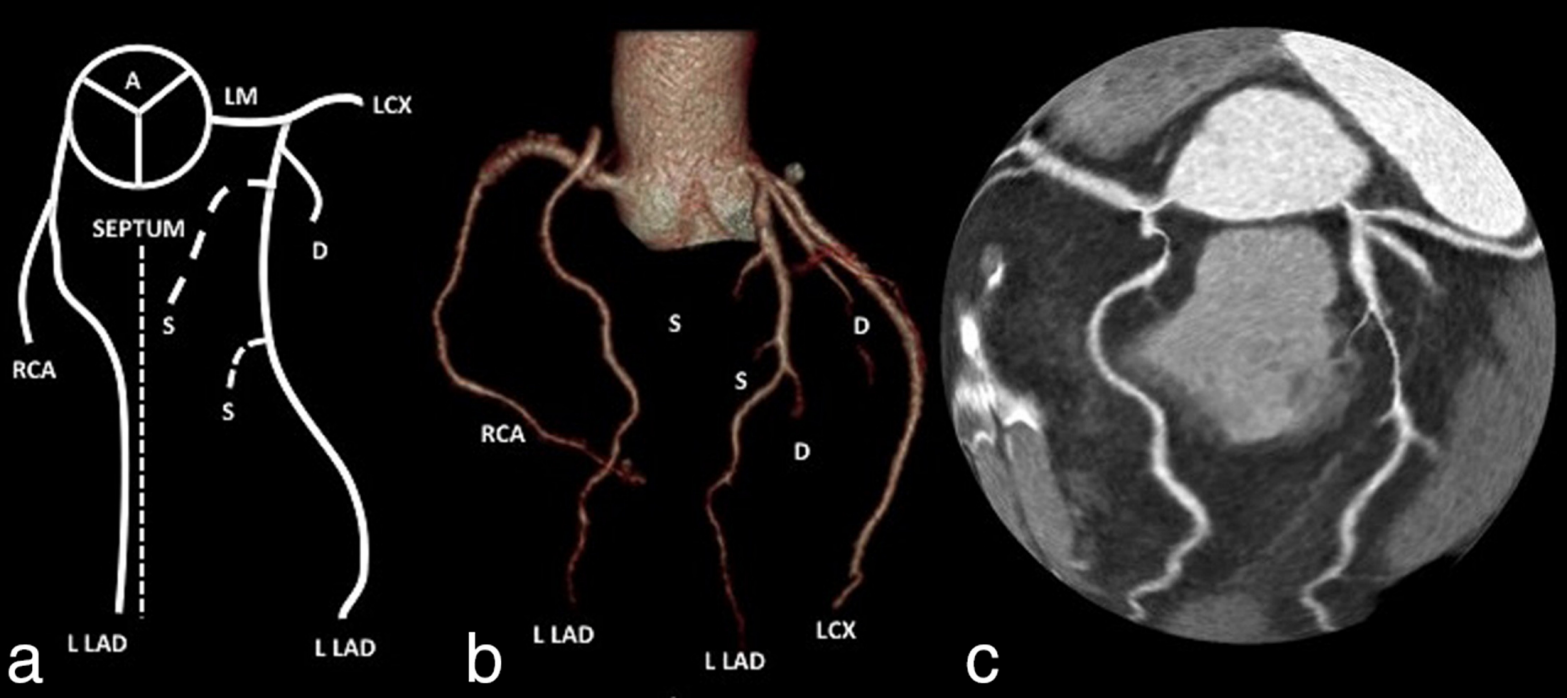
(a–c) Schematic (a), 3D volume rendering (b) and (c) 3D globe view illustration of our novel dual LAD. Dashed lines indicate the AVIS septum. A, ascending aorta; AVIS, anterior interventricular sulcus; D, diagona1 coronary artery; LCX, left circumflex artery; L LAD, long left anterior descending artery; LM, left main coronary artery; RCA, right coronary artery; S, septal coronary artery; S LAD, short left anterior descending artery.

**Figure 3.**
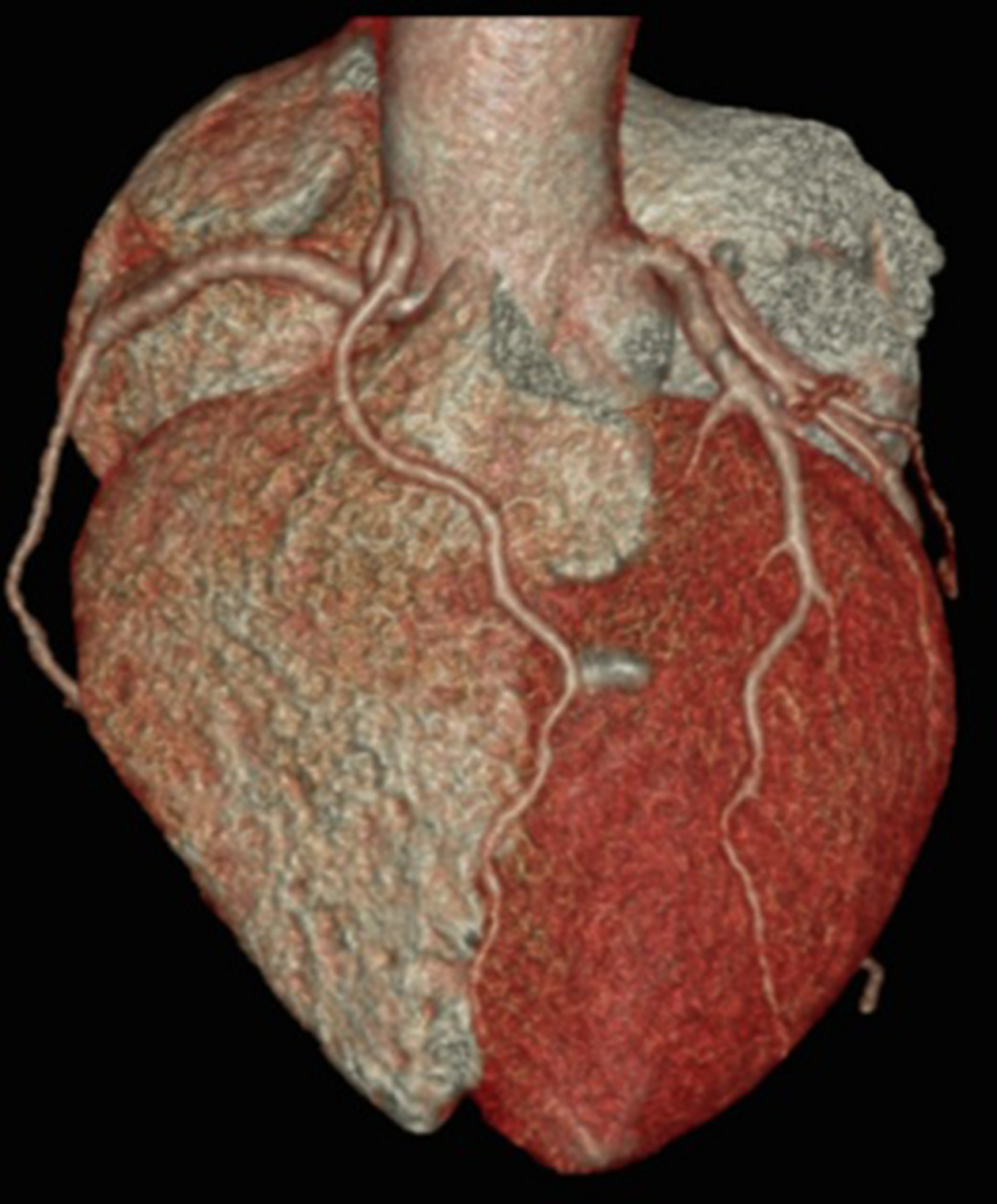
3D volume rendering of the heart with the pulmonary artery origin digitally excised showing the course of the S LAD along the epicardial surface of the LV reaching the apex, and the L LAD along the epicardial surface of the RV and into the distal AIVG. A, ascending aorta; D, diagona1 coronary artery; L LAD, long left anterior descending artery; LCX, left circumflex artery; LM, left main coronary artery; LMCA, left main coronary artery; LV, left ventricular; RCA, right coronary artery; RV, right ventricular; S, septal coronary artery; S LAD, short left anterior descending artery.

We present a case of a 51-year-old male who presented with atypical chest pain. As part of his workup and coronary artery disease (CAD) risk stratification, he underwent a Coronary CT angiography (CCTA) scan at our institution. The “short” LAD was not short since it branched out of the LMCA and coursed along the epicardial surface of the left ventricular anterior wall all the way to the apex of the heart. A long LAD branches out of the RCA and courses along the epicardial surface of the right ventricular and into and along the distal AIVG. In our patient, the long LAD joined the AIVG at its mid part. Both LAD arteries were similar in length (Figures 2 and 3).

### Technical method and discussion

With the patient positioned supine and arms placed above his head, electrocardiogram-gated cardiac/coronary CTA was performed using a 256-MDCT scanner (Philips iCT, Philips Healthcare, Amsterdam, The Netherlands). Anteroposterior and lateral scout scans were performed, with a scan range from the apex of the chest to the costophrenic angle. Scan parameters were as follows: detector width of 256 × 0.625 mm, pitch of 0.2:1 ratio, rotation time of 0.27 s, 100 kVp, 200 mA, with *z*-axis modulation, and scanning time of 2.1 s. A caudocranial scan direction was employed.

### Contrast medium administration

Via a 22 gauge venous catheter, placed in the right brachiocephalic vein, contrast media (CM) was injected with an automated dual-barrel power injector (Optivantage, Mallinckrodt, Cincinnati). Right-sided venous access was used in this study because it provides a uniform opacification incontrast to the heart; with the least possible dilution. Hence, this approach promotes optimal image quality coupled with reduced contrast volumes.^5,6^ Both the contrast media and saline injection rates were 4.5 ml s^−1^.

### Contrast bolus geometry

**Figure 4.**
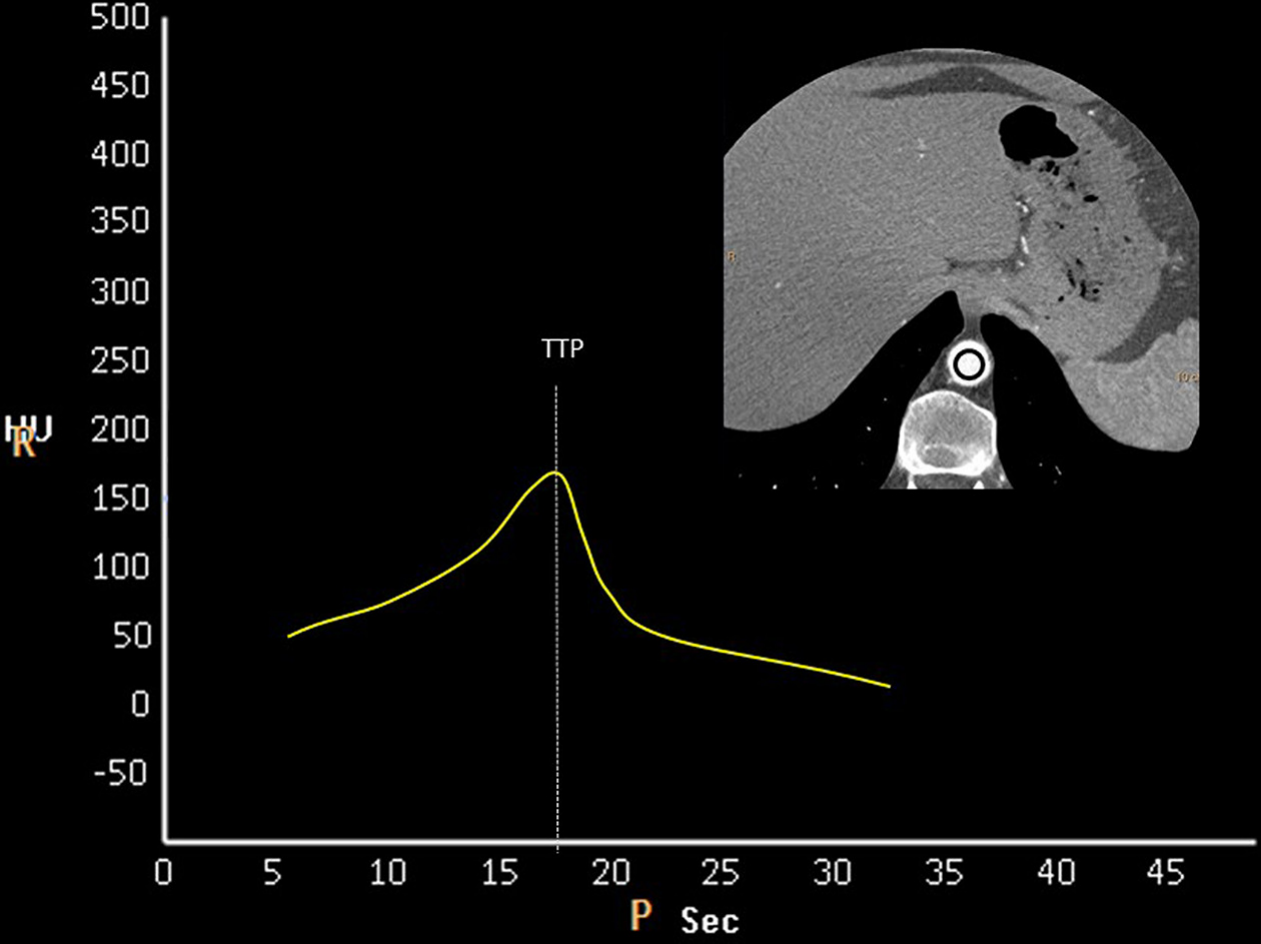
The timing bolus is used to determine the TTP of the descending thoracic aorta. Therefore, in this case study, we synchronized our CTA acquisition when we reached peak contrast opacification in the descending thoracic aorta which accounts for maximal filling of the RCA, dual LAD and LCX. CTA, CT angiography; HU, Hounsfield units; LCX, left circumflex artery; LAD, left anterior descending; RCA, right coronary artery; TTP, time to peak.

Bolus geometry is an opacification pattern measured in the region of interest.^7^ and plotted on a time (s)/attenuation Hounsfield units curve; after an intravascular injection of contrast material. This technique was employed where the region of interest was plotted inside the abdominal aorta; at the level of the aortic hiatus. The procedure comprised of a small amount of contrast material (5 mL); injected at the same rate as that of the main bolus. region of interest assessed the time to peak and determined the arteriovenous circulation time for the thoracic vasculature at the level of the aortic hiatus (Figure 4).

### Synchronisation between contrast media administration and scanner acquisition

**Figure 5.**
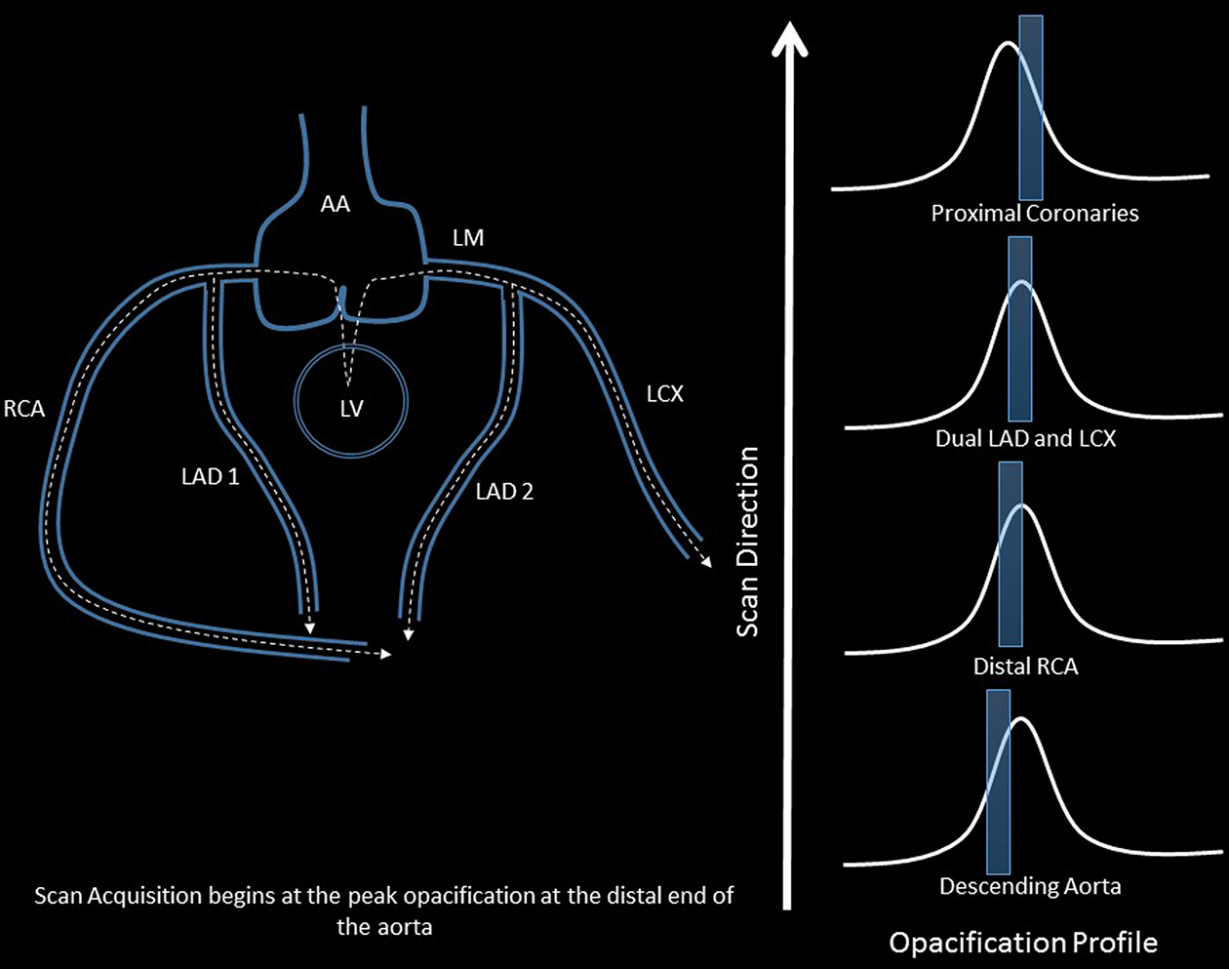
This image demonstrates the optimal time to begin the CTA acquisition relative to the contrast media opacification profile. The dotted lines with the arrows demonstrate the arterial blood flow direction, whereas the scan acquisition is in the caudocranial direction (long white arrow). The opacification profiles demonstrate the scan phase relative to the contrast opacification curve at each anatomical location. The first curve demonstrates the scan acquisition box at the level of descending aorta, whereas the last curve (relative to the end of the scan direction), shows the proximal coronary artery scan phase during the opacification profile. AA, ascending aorta; CTA, CT angiography; LAD, left anterior descending artery; LCX, left circumflex artery; LM, left main coronary artery.

To synchronize data acquisition with optimal arterial opacification, it has been recommended that scan direction during CTA should be in the opposite direction of CM flow during CTA.^5,6,8^ During CTA, it is feasible to scan at a faster rate than that of CM traversing the vessel. A drawback to faster scan acquisitions is poor arterial opacification, particularly when antegrade blood flow from the brachiocephalic trunk to the coronary arteries exists. Such pathological processes cause turbulence of blood flow before, within and after the origin of the ascending aorta, resulting in a slowing down of the passage of contrast associated with the antegrade blood flow from the origin of the ascending aorta to the distal segments of the coronary arteries. Even though there are clear limitations in the literature regarding the impact of fast scan times and associated contrast/blood flow dynamics, a practical solution to overcome such limitation is to measure the opacification peak of the descending thoracic aorta at the distal segment. Once these data are available, the exact contrast/blood flow dynamics can be predicted irrespective of blood flow dynamics. Therefore, optimal synchronisation between blood/contrast media flow with a caudocranial CT scan direction, achieves peak opacification throughout the entire hypoplastic ascending aorta and coronary arteries (Figure 5).

### Image reconstruction

The following parameters were set: standard reconstruction of axial images at 0.625 mm slice width, reconstruction interval of 0.5 mm, field of view of 180 × 180 mm and an iterative reconstruction technique software (iDose^4^; Philips Healthcare, Cleveland, OH, USA) with a window width and level of 420 and 65, respectively. The electrocardiogram-gated scan reconstruction interval, with the least motion artefacts, was determined by reconstructing a slice at the mid segment of the ascending aorta in 2% steps from 35% to 75% of the R–R interval. For diagnostic interpretation, reconstruction of the CTA images was used; where a time point with the least motion artefact was located at the mid segment of the ascending aorta (48%).

## Discussion

Dual LAD may be associated with congenital heart disease such as tetralogy of Fallot and transposition of the great arteries; or it may simply be an incidental finding at coronary angiography, or CCTA as in our patient’s case. The Type 4, dual LAD anomaly, is extremely rare and is reported in less than 0.2% of patients undergoing invasive coronary and/or CCTA. In this pattern, a short LAD usually branches out of the LMCA and courses along the proximal AIVG.

Dual LAD anomaly is associated with a benign course. However, recognising this anomaly is of utter importance in the context of an existent coronary artery disease since it is sometimes difficult to differentiate this anomaly from a total mid or distal LAD occlusion on coronary angiography.

It may perhaps be observed that identifying the coronary tree and anomalies can be difficult with coronary angiography owing to poor spatial information as to the origin, courses and terminations of the coronary arteries in relation to the different heart structures. Nevertheless, conventional angiography is unable to demonstrate the vascular tree as does coronary CTA since conventional angiography may not demonstrate critical anomalies i such as when an anomalous artery courses in between the aorta and the right ventricular outflow tract (i.e. Type 6) in which case there is a significant risk of myocardial ischaemia and sudden death. Finally, CCTA is essential whenever dual LAD anomaly is detected on conventional angiography since it demonstrates the relationship between the coronary artery tree with heart structures that results in prompt identification of higher risk types.

## Conclusions

Evaluation of complex coronary angioarchitecture in patients with congenital coronary anomalies is considerably improved with CTA.

## Learning Points

1. Patient-specific contrast media admnistration techniques improve the visualisation of coronary and thoracic vasculature during CTA.
2. Matching contrast mediadelivery with altered blood flow enhances the visulisation of dual LAD during CTA
3. Increased reader confidence is associated with optimal image quality

## Consent

Written informed consent for the case to be published (incl. images, case history and data) was obtained from the patient(s) for publication of this case report, including accompanying images.
